# Whole-brain background-suppressed pCASL MRI with 1D-accelerated 3D RARE Stack-Of-Spirals readout

**DOI:** 10.1371/journal.pone.0183762

**Published:** 2017-08-24

**Authors:** Marta Vidorreta, Ze Wang, Yulin V. Chang, David A. Wolk, María A. Fernández-Seara, John A. Detre

**Affiliations:** 1 Department of Neurology, University of Pennsylvania, Philadelphia, Pennsylvania, United States of America; 2 Department of Radiology, University of Pennsylvania, Philadelphia, Pennsylvania, United States of America; 3 Department of Radiology, Temple University, Philadelphia, Pennsylvania, United States of America; 4 Center for Cognition and Brain Disorder, Hangzhou Normal University, Hangzhou, Zhejiang Province, China; 5 Department of Radiology, Clínica Universidad de Navarra, Pamplona, Navarra, Spain; Lee Kong Chian School of Medicine, SINGAPORE

## Abstract

Arterial Spin Labeled (ASL) perfusion MRI enables non-invasive, quantitative measurements of tissue perfusion, and has a broad range of applications including brain functional imaging. However, ASL suffers from low signal-to-noise ratio (SNR), limiting image resolution. Acquisitions using 3D readouts are optimal for background-suppression of static signals, but can be SAR intensive and typically suffer from through-plane blurring. In this study, we investigated the use of accelerated 3D readouts to obtain whole-brain, high-SNR ASL perfusion maps and reduce SAR deposition. Parallel imaging was implemented along the partition-encoding direction in a pseudo-continuous ASL sequence with background-suppression and 3D RARE Stack-Of-Spirals readout, and its performance was evaluated in three small cohorts. First, both non-accelerated and two-fold accelerated single-shot versions of the sequence were evaluated in healthy volunteers during a motor-photic task, and the performance was compared in terms of temporal SNR, GM-WM contrast, and statistical significance of the detected activation. Secondly, single-shot 1D-accelerated imaging was compared to a two-shot accelerated version to assess benefits of SNR and spatial resolution for applications in which temporal resolution is not paramount. Third, the efficacy of this approach in clinical populations was assessed by applying the single-shot 1D-accelerated version to a larger cohort of elderly volunteers. Accelerated data demonstrated the ability to detect functional activation at the subject level, including cerebellar activity, without loss in the perfusion signal temporal stability and the statistical power of the activations. The use of acceleration also resulted in increased GM-WM contrast, likely due to reduced through-plane partial volume effects, that were further attenuated with the use of two-shot readouts. In a clinical cohort, image quality remained excellent, and expected effects of age and sex on cerebral blood flow could be detected. The sequence is freely available upon request for academic use and could benefit a broad range of cognitive and clinical neuroscience research.

## Introduction

Arterial Spin Labeled (ASL) perfusion MRI [1,2] utilizes magnetically labeled arterial blood water as an endogenous tracer, providing non-invasive, quantitative, repeatable perfusion measurements in units of ml/min/100g of tissue. ASL is primarily used in brain to measure cerebral blood flow (CBF), but can also be used to measure perfusion in other organs. Tissue perfusion provides fundamental information about both cerebrovascular integrity and regional neural activity, based on the tight coupling between regional CBF and glucose metabolism [3].

Achieving whole-brain CBF maps with high sensitivity, high spatial resolution and high temporal resolution is needed to study dynamic activity changes. However, this has remained challenging in ASL ever since its inception. The main limitation of ASL is its low intrinsic signal-to-noise ratio (SNR). This is mainly due to two reasons: the small amount of labeled arterial blood available per tissue volume (1–2%) [4], and the T_1_ decay of the labeled signal from the labeling location to the imaging tissue. Low SNR limits image resolution, which in turn results in partial volume effects that translate into image blurring.

Although many labeling and readout strategies exist, a recent consensus has been reached within the ASL community towards the use of pseudo-continuous labeling (pCASL) [5], combined with background suppression [6,7] and segmented 3D readouts to maximize ASL SNR [8]. While segmented readouts can be employed to increase the spatial resolution and/or decrease image blurring in exchange for temporal resolution [9–11], they are typically sub-optimal for functional studies, where high temporal sampling is often required. Single-shot 3D acquisitions provide higher temporal resolution, but have typically been limited to a through-plane voxel resolution of 6-7mm for whole-brain imaging so as to avoid severe image blurring due to T_2_ decay. An ideal ASL perfusion MRI methodology would provide the versatility to optimize either spatial or temporal resolution, based on the application.

The use of RF coil arrays and parallel imaging techniques [12] provides a means towards achieving higher temporal resolutions. With parallel imaging, both readout length and SAR deposition can be reduced by leveraging the spatially localized sensitivity of each component coil to recover the non-acquired k-space data. Despite the reduced sampling time with increasing acceleration factors, previous work has found no quality loss in the perfusion images with two-fold accelerations in 2D echo-planar imaging ASL data [13] due to the increased perfusion signal achieved by the shortened TE and readout time.

In this work, parallel imaging was implemented aIong the partition-encoding (k_z_) direction in a pCASL sequence with background suppression and 3D RARE Stack-Of-Spirals readout, using a 1D GRAPPA [14] approach to reconstruct the missing partitions and to achieve whole-brain single-shot imaging with 3.75mm isotropic resolution. Although in-plane acceleration has been implemented in 3D GRASE readouts to reduce TE in ASL imaging [15], this strategy is sub-optimal for reducing readout length, and the implications of the acceleration have not been thoroughly studied. Recent work has combined 3D RARE Stack-Of-Spirals readouts with in-plane acceleration and compressed sensing [16] or 3D acceleration [17], demonstrating their potential for model-based dynamic ASL imaging or high-resolution perfusion imaging. However, these strategies currently involve offline, iterative reconstruction methods that are highly computationally demanding, hindering their implementation in clinical practice, while GRAPPA-based reconstruction routines are readily available in all scanner platforms for online reconstruction of the images.

In this paper, we presented a new ASL sequence, that combines a background suppressed pCASL labeling scheme and a 1D accelerated 3D readout with online reconstruction, as an extension of our previously published pCASL sequences [11,18]. This sequence supports both acceleration and multiple-segment acquisition (i.e. multi-shot readout) and thus provides a versatile framework for users to optimize their protocols based on the desired temporal and spatial resolution of the images.

First, the theoretical benefits of 1D acceleration were assessed by simulating the point spread function (PSF) to estimate achievable effective voxel sizes with and without acceleration. Then, the functionality of this ASL approach was evaluated in three different set-ups: In Study 1, the sensitivity of the single-shot, 1D accelerated ASL sequence to detect dynamic CBF changes was assessed using a task activation paradigm. Both non-accelerated and two-fold accelerated versions of the sequence were evaluated in healthy volunteers during a motor-photic task, and the performance was compared in terms of temporal SNR (tSNR), GM-WM contrast, and statistical significance of the detected activation. In Study 2, single-shot was compared to multi-shot acquisition to illustrate how trade-offs in spatial and temporal resolution affect ASL image quality. Specifically, single-shot 1D-accelerated imaging was compared to a two-shot version to assess benefits of SNR and spatial resolution for applications in which temporal resolution is not paramount. Finally, Study 3 tested the efficacy of this approach in a clinical population undergoing a multimodal MRI protocol, analogous to a typical radiological MRI examination, by applying the single-shot 1D-accelerated version to a cohort of elderly volunteers.

## Materials and methods

### Description of the ASL sequence

A sequence with pseudo-continuous labeling, background suppression and 3D RARE Stack-Of-Spirals readout with optional through-plane acceleration was implemented for this study. At the beginning of the sequence, gradients were rapidly played with alternating polarity to correct for their delay in the spiral trajectories [19], followed by two preparation TRs, to allow the signal to reach the steady state. A non-accelerated readout was played during the preparation TRs, in order to obtain a fully sampled k-space dataset, used for calibration of the parallel imaging reconstruction kernel, needed to reconstruct the skipped k_z_ partitions in the accelerated images.

#### Labeling

The pCASL pulse consisted of a train of selective RF pulses (Hanning window, B1_average_ = 1.87 μT, duration = 500 μs, spacing = 500 μs, G_average_ = 1 mT/m, G_maximum_/G_average_ = 8). The phase of each RF pulse was incremented by a constant amount to maintain coherence with the flowing spins under the non-zero mean gradient. For the control, the RF phase alternated from 0 to 180° between pulses. Identical gradient waveforms were used for label and control acquisitions. The position of the labeling plane was set individually for each subject upon visualization of the angiograms and maintained across runs. The location was chosen so that both internal carotid and vertebral arteries were oriented as perpendicular as possible to the inversion plane.

#### Background suppression

An optimized background suppression scheme was added to the sequence to suppress the static tissue signal. The scheme consisted of a presaturation module at the beginning of each TR, followed by a set of adiabatic inversion pulses distributed during the labeling/post-label delay (PLD) times, with timings optimized to suppress the static magnetization to 10% of its equilibrium value at the start of readout.

#### Readout

A 3D RARE Stack-Of-Spirals readout was employed, consisting of a 90° excitation RF pulse followed by a series of 180° refocusing RF pulses. Each k_z_ partition was divided into two spiral interleaves, with each interleaf acquired between a separate pair of refocusing RF pulses. The spiral readout trajectories were generated numerically (King et al., 1995), assuming maximum gradient amplitude and slew rate of 36 mT/m and 120 mT/m/ms, respectively, and receiver bandwidth = 400 kHz. The spiral calculation code was derived from Dr. Brian Hargreaves’ code (http://mrsrl.stanford.edu/~brian/vdspiral/). To avoid FOV aliasing artifacts, the Nyquist criterion was applied along the radial direction. A centric-encoding scheme was applied for partition order acquisition. When the sequence was applied in single-shot mode, the two spiral interleaves were acquired sequentially in the echo train, while if a two-shot mode was applied, each spiral interleaf was acquired in a separate TR or shot.

Images were acquired with 3.75 mm isotropic resolution, FOV = 240×240×128 mm³, 34 nominal partitions with 5.9% oversampling and slice PF = 5/8 (single-shot) or 6/8 (two-shot), in-plane matrix = 64x64, effective TE = 10.3 ms. Total readout times were 741, 390 and 231 ms for single-shot non-accelerated, single-shot accelerated and two-shot accelerated, respectively. A TR of 4.5 s was used for the non-accelerated case, and a TR of 4 s for the accelerated cases.

#### 1D acceleration

**Fig 1:**
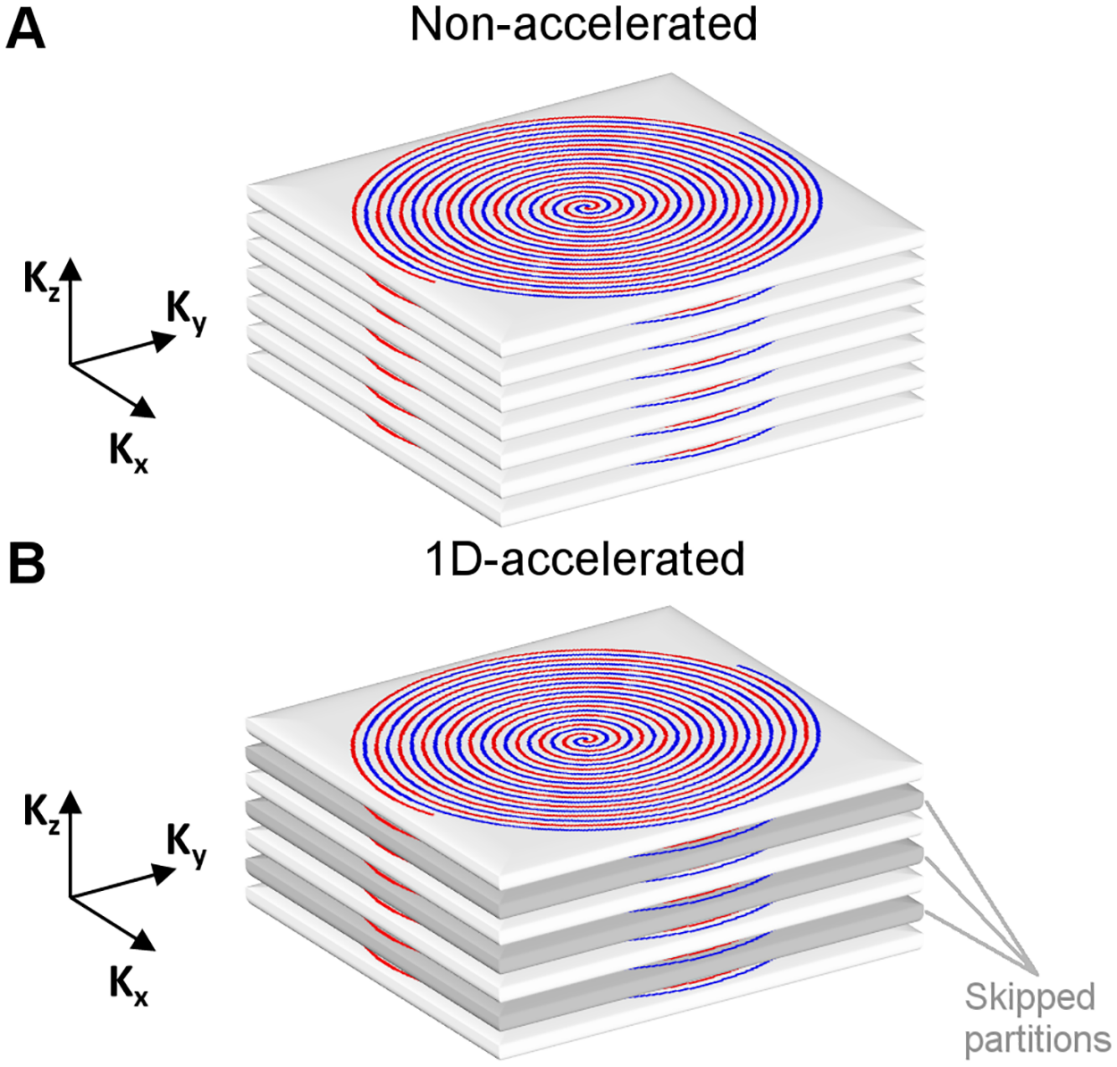
Illustration of the (A) non-accelerated and (B) 1D-accelerated 3D RARE Stack-Of-Spirals readout employed. Two spiral interleaves were used to sample each partition (shown in red and blue). In the 1D-accelerated readout, every other k_z_ partition is skipped due to undersampling (highlighted in gray).

Acceleration was implemented in the k_z_ direction (Fig 1). Acquired partitions were selected starting from the central partition (k_z_ = 0), and moving towards outer frequencies in multiples of 2 (k_z_ = ± 2n), resulting in an acquisition with an acceleration factor of 2.

The 1D acceleration accounted for a 15% reduction of the deposited RF energy in the protocol employed in the single-shot form, which rose up to 24% when combined with two-shot.

#### Data reconstruction

The spiral trajectories were corrected prior to reconstruction by estimating the gradient time delays [19]. Spiral k-space data were then regridded to Cartesian coordinates using the standard regridding method with a Kaiser-Bessel window kernel [20], and a Voronoi diagram to correct non-uniform sampling density.

In the accelerated datasets, the missing partitions were recovered from the nearest neighboring partitions using a GRAPPA-like reconstruction approach [14]. This autocalibrated method is used to reconstruct uncombined coil channel images from each channel in the array, without the need of explicitly obtaining coil sensitivity maps. In this method, the missing data from each partition and channel coil are reconstructed by means of a linear combination of the neighboring data from all channels. To that end, first a 4x1 kernel was calculated from the calibration data acquired at the beginning of the scan. The kernel was then used to estimate the channel data for each missing partition due to undersampling in the subsequent acquisitions.

The POCS (Projection Onto Convex Sets) algorithm [21] was used for slice partial Fourier reconstruction. Finally, the Fourier Transform was used to transform the data into image space. The resulting uncombined coil channel images were combined by means of sum of squares reconstruction.

The reconstruction pipeline was first carried out offline using Matlab scripts and then implemented in the Siemens Ice online reconstruction platform.

### Scanning protocol

Three studies were conducted in order to evaluate the performance of the perfusion sequence. All studies were approved by our internal review board. Adult, healthy controls were recruited to participate in the studies, defined by the inclusion criteria of being at least 18 years old and not having a history of neurologic or psychiatric disorders. The demographic information of the recruited participants is summarized in S1 Table.

In Study 1, non-accelerated and accelerated versions of the sequence were compared during the execution of a functional activation paradigm. In Study 2, single-shot and two-shot versions of the accelerated sequence were acquired during rest. Lastly, in Study 3, perfusion data were acquired on an elderly cohort using the single-shot, accelerated sequence. All data are available from the OpenfMRI database (https://openfmri.org), accession numbers ds000234, ds000235 and ds000236.

For each participant, first a high-resolution anatomical T_1_-weighted image was acquired with a magnetization prepared rapid gradient echo (MPRAGE) sequence, followed by a Time-Of-Flight (TOF) angiogram acquired at the base of the brain. The imaging parameters employed for both acquisitions in each study are detailed in the S1 File.

#### Study 1

The study was conducted on a 3 Tesla Siemens Trio, using a 32-channel head array.

Five healthy volunteers (1 female, mean age ± standard deviation (SD) = 35 ± 11.5 years, range = 27–55 years) participated in the study, after signing a written informed consent.

Subjects underwent two perfusion runs, in which functional data were acquired with the non-accelerated and the accelerated version of the sequence, in pseudo-randomized order, during a visual-motor activation paradigm. During each run, 3 resting blocks alternated with 3 task blocks, with each block comprising 8 label-control pairs (72 s and 64 s for the non-accelerated and accelerated sequence versions, respectively). During the resting blocks, subjects were instructed to remain still while looking at a fixation cross. During the task blocks, a flashing checkerboard was displayed and subjects were asked to tap their right-hand fingers while looking at the center of the board. Labeling and PLD times were 1.5 and 1.5 s. In addition, four M0 images with long TR and no magnetization preparation were acquired per perfusion run for CBF quantification purposes.

#### Study 2

The study was conducted on a 3 Tesla Siemens Prisma, using a 64-channel head array.

Four healthy volunteers (2 female, mean age ± SD = 43.0 ± 20.8 years, range = 21–65 years) participated in the study, after signing a written informed consent.

Subjects were instructed to remain still and awake, while resting perfusion data were acquired using either 1-shot or 2-shot 1D-accelerated readout. 64 and 32 label-control images were acquired, respectively, during a total scan time of 5 min. Labeling and PLD times where 1.8 and 1.8 s. Two M0 images with long TR and no magnetization preparation were acquired per run for CBF quantification purposes.

#### Study 3

The study was conducted on a 3 Tesla Siemens Prisma, using a 64-channel head array.

Eighteen elderly volunteers (12 female, mean age ± SD = 74.6 ± 8.5 years, range = 61–93 years) participated in the study, after signing a written informed consent. All participants were recruited from the Penn Memory Center, a National Institute of Aging supported Alzheimer’s disease center. Inclusion criteria are detailed in the S1 File.

Resting perfusion data were acquired with a 1-shot 1D-accelerated readout for a total scan duration of 5 min, with labeling and PLD times of 1.5 and 1.5 s. Two M0 images with long TR and no magnetization preparation were acquired per run for CBF quantification purposes.

### Data processing

ASL data were preprocessed using the FMRIB software library (FSL), version 5.0.8 (http://www.fmrib.ox.ac.uk/fsl/), and custom scripts in Matlab (Mathworks, MA, USA).

First, for each acquisition the raw label, control and M0 images were realigned to the first image of the series. Six rigid-body motion parameters were estimated with MCFLIRT. Before applying the transformation to the images, the spurious effect of the label-control intensity differences was removed from the motion parameters using linear regression [22]. Then, images were registered to the anatomical dataset using FLIRT. M0 images were smoothed with a 4 mm Full-Width at Half-Maximum (FWHM) Gaussian kernel and averaged to create a mean M0 image. Perfusion images were then obtained by pair-wise subtraction, and converted into CBF maps by means of the single-compartment model [23], in units of ml of blood per min perfused in 100g of tissue. A mean perfusion map and a mean CBF map were then calculated per run by averaging all individual perfusion and CBF maps, respectively.

T_1_-weighted anatomical images were segmented into gray matter (GM) and white matter (WM) tissue probability maps using the New Segment tool in SPM, version 8 (Wellcome Trust Center for Neuroimaging, University College London, UK). GM and WM masks were derived using probability thresholds 0.75 and 0.9, to avoid voxels contaminated by partial volume effects. Whole-brain masks were also derived for each subject, upon combination of the GM and WM tissue maps (combined GM+WM threshold = 0.25) and filling in the inner CSF voxels.

### Point spread function simulations

Greater voxel size images provide higher SNR, but also show typically lower between-tissue contrast and more severe partial volume effects. The width of the PSF can be used as an indicator of the effective voxel size of an image sampled with a specific readout. Therefore, to assess the effect of acceleration on the effective image resolution in the slice direction, and thus, on the image SNR, PSF simulations were carried out for each sequence version. To this end, the signal amplitudes for each k_z_ plane were calculated, assuming the following arterial blood relaxation times: T_2,blood_ = 186 ms [24], T_2*,blood_ = 73 ms [25]. After reordering, the modulation transfer functions (MTF) were obtained, from which the PSFs were computed by direct Fourier Transform. To evaluate the effective voxel resolutions, the FWHM of the PSFs were compared across readouts.

### Data analysis

In all studies, whole-brain, GM and WM mean perfusion and CBF values were extracted from the perfusion and CBF maps for each sequence version, as described in earlier work [26], and used to derive a GM-WM contrast ratio measure, computed as the ratio of the mean GM to WM CBF values. Temporal SNR maps were also derived from each dataset to extract mean GM and WM voxel tSNR values, by dividing for each voxel the mean of its perfusion time series over its standard deviation.

The image noise level was also estimated in the mean perfusion maps by carefully selecting a region outside the brain devoid of artifacts, and computing the SD across the voxels within that region. Upon inspection, this region was selected as the rightmost sagittal plane, excluding the corners. The value obtained was corrected for magnitude reconstruction and multiple averaging [27].

In Study 1, statistical differences in CBF and tSNR between readouts were assessed by means of a paired permutation test (N_permutations_ = 32 for N = 5, p < 0.0313). The ability of each sequence version to detect functional activation was also evaluated at both subject and group levels. At the individual level, data was analyzed using the general linear model [28] by fitting the data into a block-design with two alternating conditions (rest and task). Individual t-maps of the comparison between task and rest were obtained for each subject and thresholded at p < 0.001 (unc.). From these maps, maximum voxel t-values were extracted at the motor and visual cortex. Finally, individual contrast (β) maps were normalized to the Montreal National Institute (MNI) template, smoothed with FWHM = 8 mm, and entered into a non-parametric one-sample t-test to derive a group activation map for each readout using a permutation test approach. Differences in the activation maps were also evaluated using an analogous analysis.

## Results

The values reported throughout this section are shown as *mean ± SD [median]*.

### Point spread function simulations

**Fig 2:**
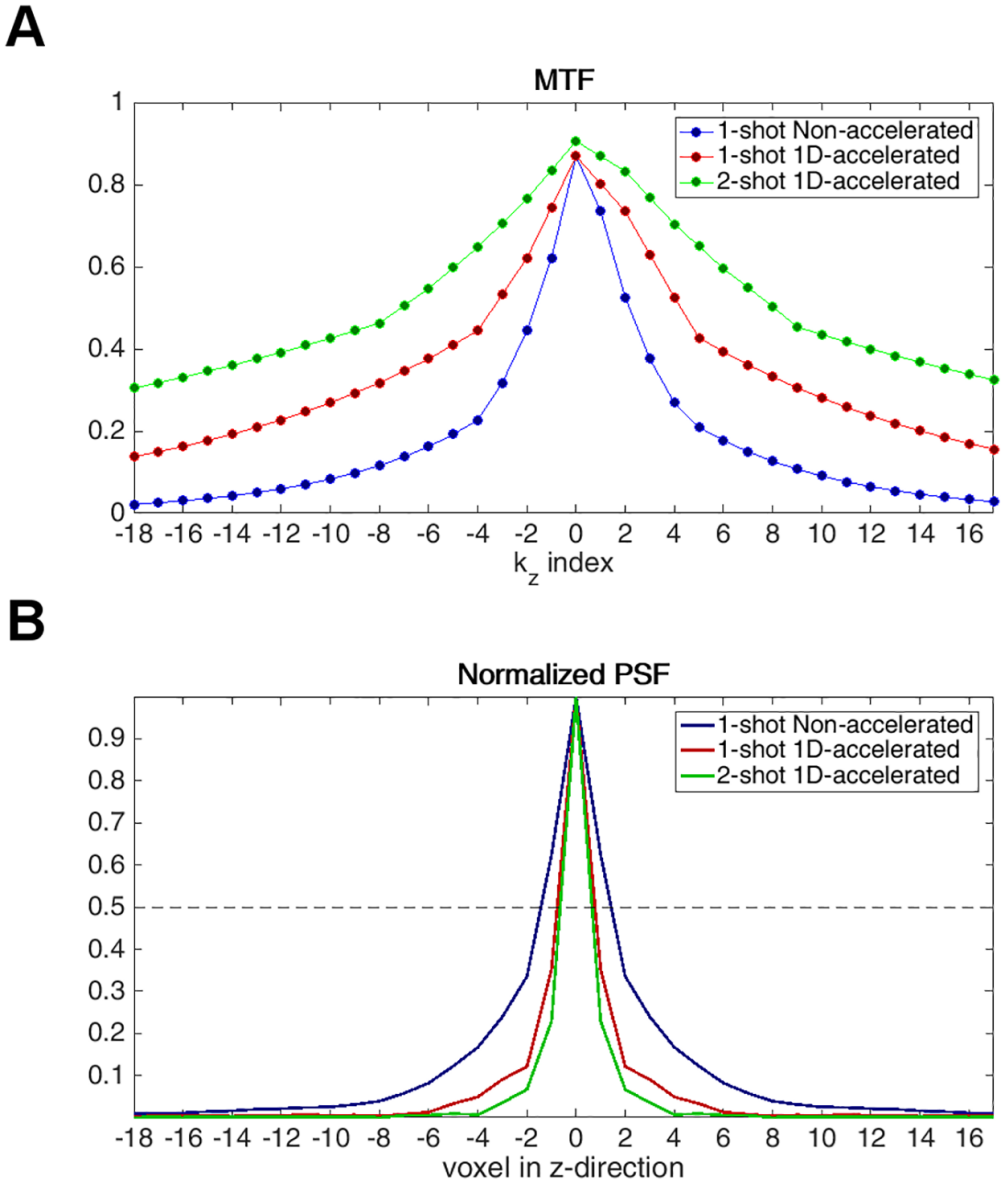
(A) Modulation transfer function (MTF) and (B) normalized point spread function (PSF) of the non-accelerated (blue) and 1D-accelerated (1-shot = red, 2-shot = green) readouts. The width of the PSF decreased from 2.86 to 1.54 (1-shot) and 1.30 (2-shot) with the use of acceleration, increasing the effective voxel resolution.

Fig 2 shows the simulated through-plane MTF and corresponding PSF for the 1-shot non-accelerated, 1-shot 1D-accelerated and the 2-shot 1D-accelerated readouts. The FWHM values of the simulated PSFs were 2.86, 1.54 and 1.30, respectively. Accelerating in the slice direction corresponded to an 84% reduction in the effective voxel resolution in this direction, and extending the acquisition to 2 shots further reduced it in 18.5%.

The area under the curve in the MTF indicates the maximum signal gain achievable at each voxel. Thus, the greater MTF areas achieved in the 1-shot and 2-shot 1D-accelerated readouts achieved by the reduced T2 decay predict maximum SNR gains of 1.93 and 2.77 with respect to the non-accelerated readout, and a 1.44 gain for going from 1-shot to 2-shot. Note that these values only constitute upper-bound limits on the maximum achievable SNR gain under the assumption of a uniformly distributed frequency spectrum, and actual values are likely to be lower due to the nature of the MR signal to be dominated by the lower frequencies, where the difference between MTFs is much smaller.

### Study 1—Non-accelerated vs 1D-accelerated readout

**Fig 3:**
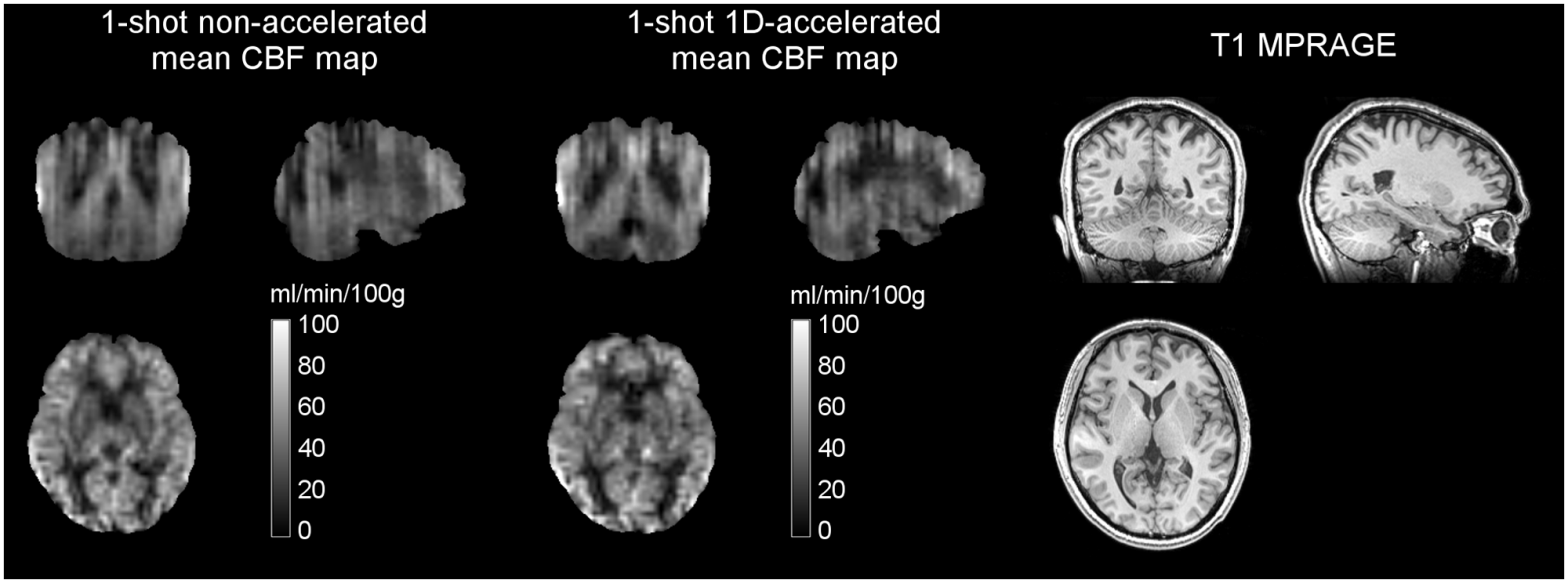
Mean CBF maps obtained in Study 1 without and with acceleration from a representative subject, in ml/min/100g, alongside the corresponding anatomical dataset. Note the reduced through-plane blurring and increase in GM—WM tissue contrast with acceleration in the coronal and sagittal views.

Whole-brain mean CBF values measured during the non-accelerated and 1D-accelerated runs were 41.17 ± 5.13 [41.00] and 39.40 ± 4.35 [38.92] ml/min/100g, respectively, with no significant differences found across runs (p = 0.1). Fig 3 shows the mean CBF maps of a representative subject obtained with the two sequences.

The noise SD measured for the non-accelerated and 1D-accelerated readouts were 0.17 ± 0.02 [0.16] and 0.23 ± 0.05 [0.21], respectively, corrected for multiple averaging. This yielded a mean increase noise factor of 1.41 ± 0.28 [1.34] across readouts. Conversely, raw accelerated data presented a mean global signal increase of 15% with respect to the non-accelerated data. Consequently, the spatial SNR was 3.81 ± 1.05 [3.67] and 3.41 ± 0.55 [3.17] for the non-accelerated and 1D-accelerated readouts, respectively, resulting in a 1.28 factor difference.

Table 1 summarizes the rest of the parameters extracted from the subjects’ data during rest. Accelerated data showed higher GM-WM CBF contrast: although no changes were observed in deep GM, WM signal appeared to decrease with acceleration, likely due to the reduced partial volume effects as a consequence of the attenuated blurring. Despite the increased background noise, no differences were observed in GM voxel tSNR (p = 0.13). WM voxel tSNR showed a decreasing trend with acceleration, consistent with the observed decrease of signal, although significance was not reached (p = 0.06).

**Table 1:** Parameters extracted from the non-accelerated and 1D-accelerated time series in Study 1, characterizing the gray matter (GM) and white matter (WM) acquired signals. Values are calculated at the voxel-level and averaged first within-tissue and finally across subjects, yielding group values given in mean ± SD [median].

*Study No*.	*Sequence type*	GM					WM					GM-WM
		Perfusion [a.u.]	M0 [a.u.(x100)]	CBF [ml/min/100g]	Temporal SNR	Spatial SNR	Perfusion [a.u.]	M0 [a.u.(x100)]	CBF [ml/min/100g]	Temporal SNR	Spatial SNR	CBF ratio
***Study 1***	**1-shot** **Non-accelerated**	7.13 ± 1.30 [7.06]	12.05 ± 0.76 [12.30]	47.43 ± 6.19 [46.12]	3.81 ± 1.05 [3.61]	4.05 ± 0.31 [4.05]	4.67 ± 0.79 [4.50]	13.63 ± 0.64 [13.96]	31.40 ± 4.48 [29.63]	2.80 ± 0.67 [2.93]	2.66 ± 0.18 [2.73]	1.51 ± 0.03 [1.52]
	**1-shot** **1D- accelerated**	8.23 ± 1.38 [8.23]	13.63 ± 0.75 [13.91]	46.62 ± 5.85 [46.97]	3.41 ± 0.55 [3.17]	3.39 ± 0.46 [3.58]	4.37 ± 0.67 [4.49]	15.05 ± 0.58 [15.21]	26.57 ± 3.55 [27.19]	1.99 ± 0.17 [2.04]	1.81 ± 0.28 [1.91]	1.76 ± 0.05 [1.75]
***Study 2***	**1-shot** **1D- accelerated**	1.65 ± 0.34 [1.70]	2.60 ± 0.16 [2.62]	59.21 ± 10.00 [61.88]	2.81 ± 0.23 [2.82]	6.00 ± 1.26 [5.76]	0.82 ± 0.13 [0.85]	2.46 ± 0.10 [2.47]	33.69 ± 5.46 [35.06]	1.70 ± 0.18 [1.71]	2.99 ± 0.46 [2.89]	1.76 ± 0.07 [1.73]
	**2-shot** **1D- accelerated**	2.01 ± 0.44 [2.08]	3.03 ± 0.21 [3.03]	62.17 ± 10.99 [65.34]	3.10 ± 0.56 [3.00]	8.74 ± 2.02 [9.36]	0.74 ± 0.12 [0.75]	2.68 ± 0.12 [2.68]	28.24 ± 4.17 [28.97]	1.44 ± 0.12 [1.41]	3.23 ± 0.56 [3.35]	2.19 ± 0.12 [2.22]
***Study 3***	**1-shot** **1D- accelerated**	1.37 ± 0.34 [1.31]	2.82 ± 0.14 [2.80]	43.58 ± 11.06 [42.73]	2.12 ± 0.61 [2.03]	4.52 ± 1.08 [4.26]	0.76 ± 0.22 [0.71]	2.92 ± 0.17 [2.89]	26.80 ± 7.72 [25.77]	1.55 ± 0.50 [1.53]	2.54 ± 0.95 [2.33]	1.66 ± 0.19 [1.69]

**Fig 4:**
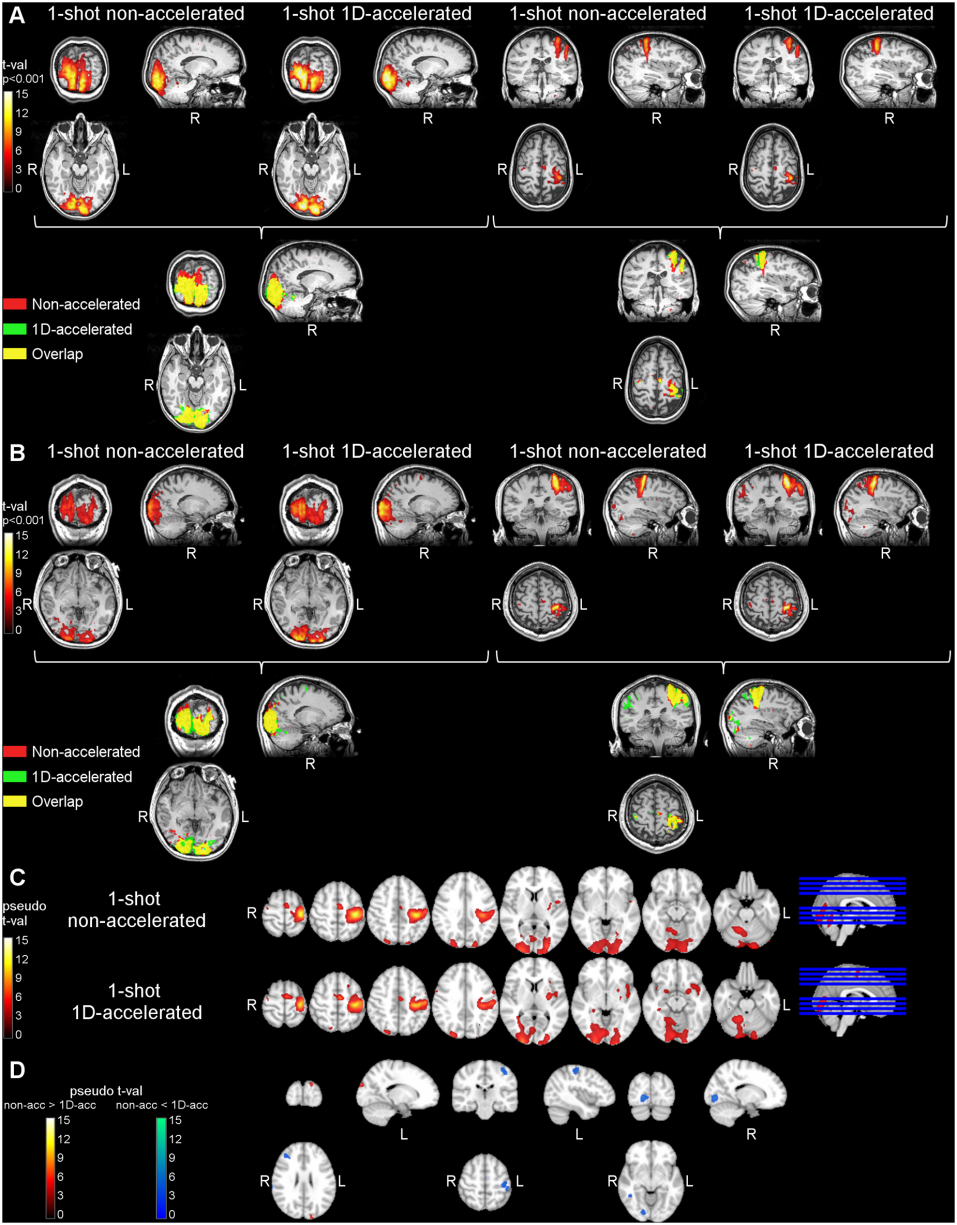
Results of the functional activation data analysis in Study 1. Panels (A) and (B) show the task>rest activation clusters for two representative subjects, obtained with the non-accelerated and the 1D-accelerated data in both visual (left) and motor (right) areas. Panel (C) shows the group activation maps obtained with each readout type. Panel (D) shows the differences in activity across readouts. Non-accelerated data only presented higher activity than accelerated data in the white matter region below the left motor cortex cluster and above the primary visual cortex cluster, likely reflecting the effects of image blurring, while 1D-accelerated data presented higher differential activation within both clusters.

The results of the functional activation analysis are summarized in Table 2 and Fig 4. All subjects showed significantly higher activity in primary visual and motor regions, including cerebellum, during the task blocks compared to the rest blocks. Table 2 gathers the maximum t-values obtained with each dataset in motor and visual cortices. Fig 4A and 4B show the activity maps obtained for two of the subjects during the non-accelerated and the 1D-accelerated runs, as well as their overlap. Fig 4C depicts the group activation maps obtained with each readout, after normalization and smoothing, and Fig 4D shows the activation differences found between readouts. Activation maps obtained with the 1D-accelerated readout showed more compact clusters, both at the individual and at the group level. When comparing Non-accelerated > 1D-accelerated, significant differences were only found in areas below the left motor cortex or above the primary visual cortex, likely reflecting the effects of blurring in the slice direction, while the accelerated data showed higher activation in the regions of interest.

**Table 2:** Maximum t-values of the activations obtained with the non-accelerated and 1D-accelerated datasets in Study 1 in primary visual and motor cortices. Values are averaged across subjects, and given in mean ± SD [median].

*Sequence type*	*Visual cortex*		*Motor cortex*
	*Left*	*Right*	*Left*
1-shot Non-accelerated	8.98 ± 3.43 [9.14]	10.17 ± 4.67 [10.21]	13.99 ± 5.59 [16.49]
1-shot 1D- accelerated	10.77 ± 2.78 [10.09]	10.77 ± 2.84 [12.09]	12.87 ± 5.11 [13.43]

### Study 2—1-shot vs 2-shot 1D-accelerated readout

**Fig 5:**
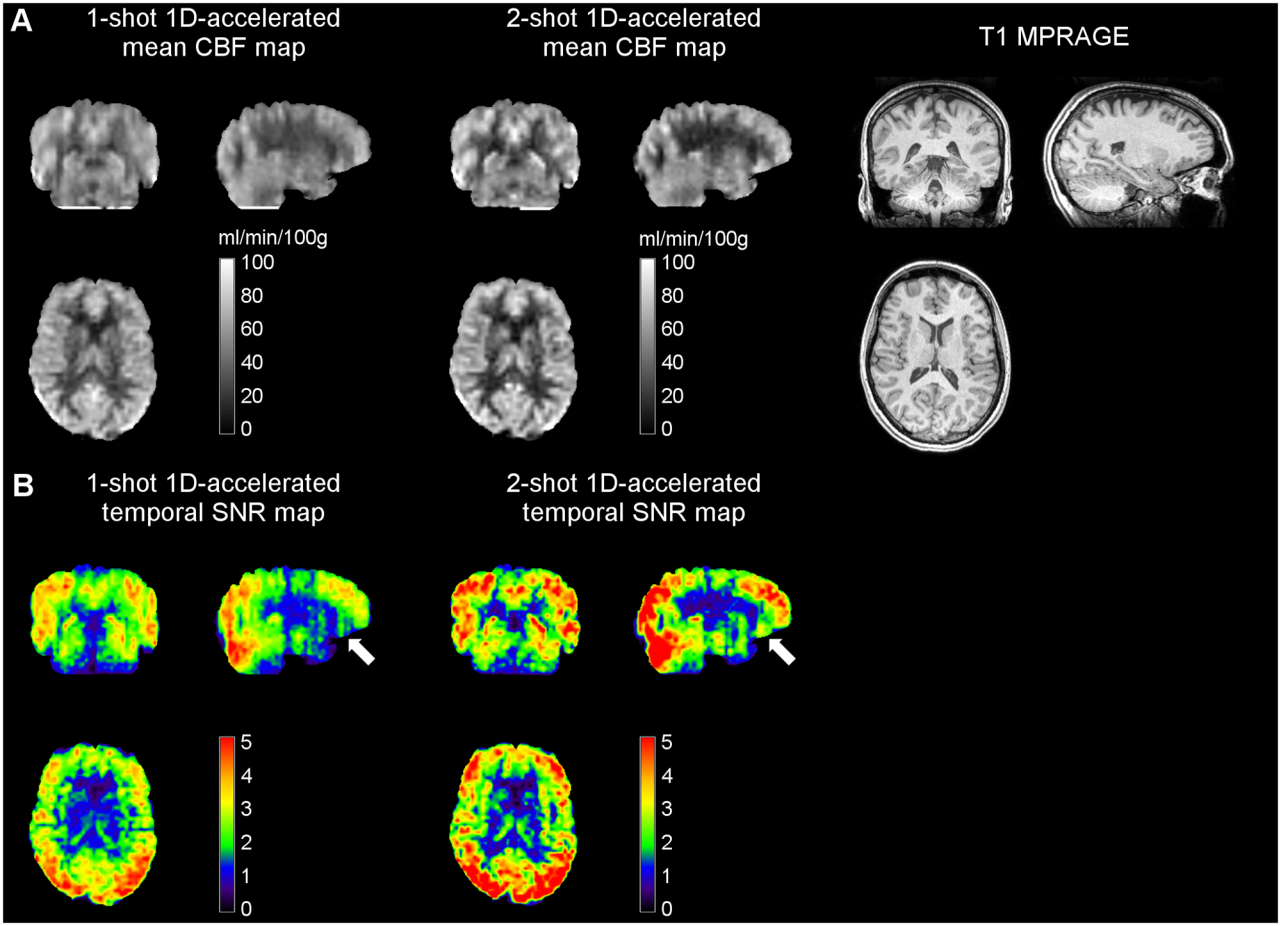
(A) Mean CBF and corresponding (B) temporal SNR maps obtained with 1-shot and 2-shot 1D-accelerated readouts in Study 2 from a representative subject, alongside the anatomical dataset. Note how the two-shot readout resulted in both an increase in SNR and a further reduction in through-plane blurring, evident in the coronal and sagittal views. White arrows point at orbitofrontal areas, which typically suffer from low SNR due to shortened T2* and T2. CBF units are in ml/min/100g.

Whole-brain mean CBF values measured in this study using the 1-shot and 2-shot 1D-accelerated runs were 46.37 ± 8.82 [48.99] and 46.19 ± 8.98 [48.58] ml/min/100g, respectively. Fig 5 shows the mean CBF maps of a representative subject obtained with the two sequences, as well as their corresponding temporal SNR maps.

A global increase in SNR was observed in the 2-shot data (spatial SNR = 5.84 ± 1.19 [6.20]) compared to the 1-shot data (spatial SNR = 4.33 ± 0.77 [4.16]), in good agreement with the gain theoretically predicted between the two readouts. A local SNR increase was also found in high-susceptibility regions with short T2 and T2* values (see white arrows in Fig 5), likely as a result of shortening the readout length. In WM, the perfusion signal was found to decrease, indicating the prevalence of some partial volume effects in the slice direction in the 1-shot data, despite the acceleration. Dividing the readout into 2 shots translated into an even narrower PSF (see *Point Spread Function Simulations* Section), increasing the GM-WM contrast ratio and the effective voxel resolution.

### Study 3—Elderly cohort

Whole-brain mean CBF values measured in this study using the 1-shot 1D-accelerated runs were 34.33 ± 8.61 [33.67] ml/min/100g. These values were lower than those found in Study 1 and Study 2 (two-sample t-test, p < 0.01), as expected due to the CBF decrease associated with age. When both sex (F = 1, M = 0) and age were entered as independent variables in a linear regression model, with CBF as dependent variable, significant effects of both factors were found (k_0_ = 48.63; β_age_ = -0.25, p_age_ = 0.002; β_sex_ = 6.81, p_sex_ = 0.026), in line with the literature [29].

**Fig 6:**
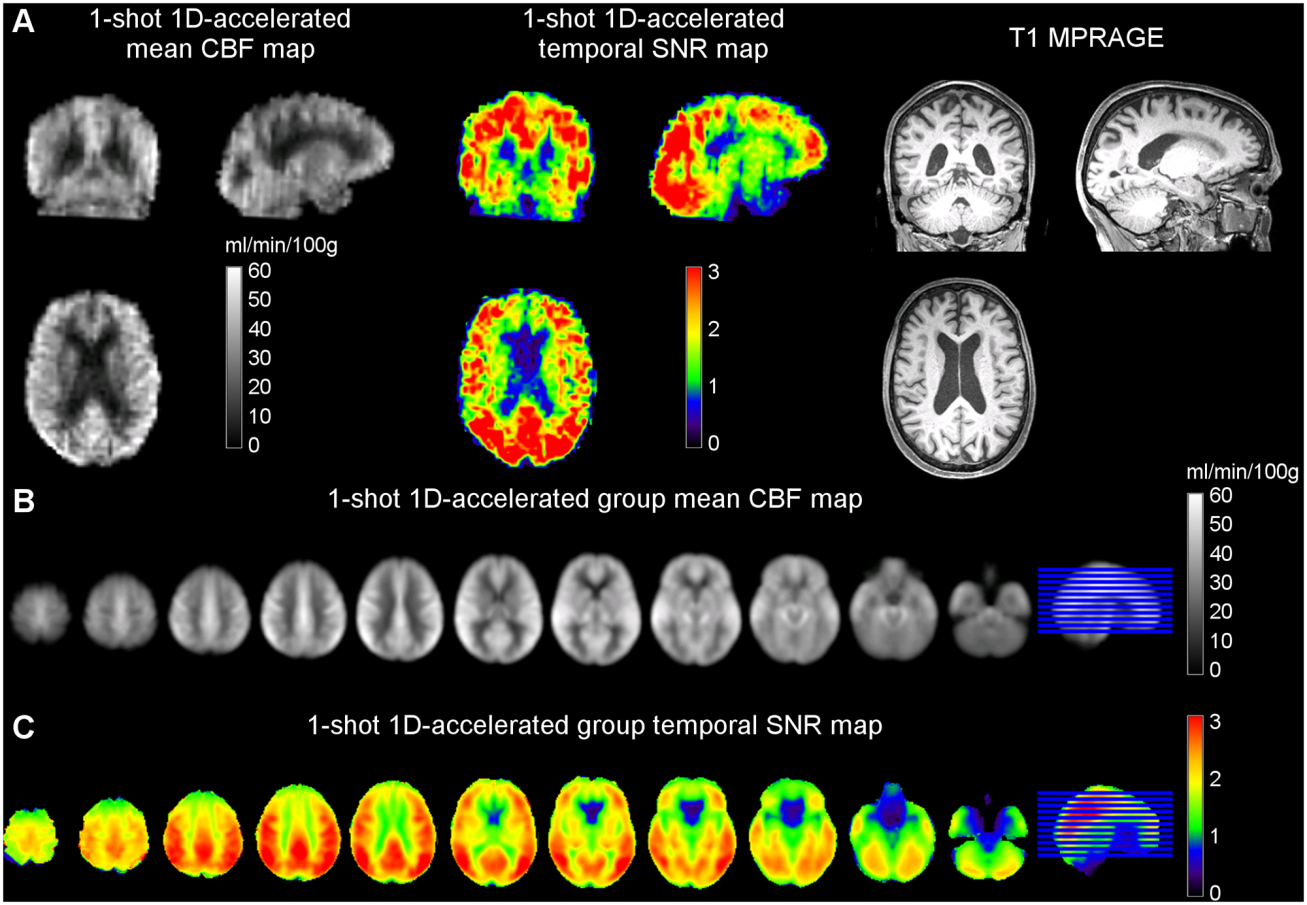
Mean CBF and temporal SNR maps of the Study 3 elderly cohort obtained from (A) a representative subject and (B) the group average after registration to MNI space and smoothing. The lowest SNR values were found in orbitofrontal regions, due to the slight sensitivity of spiral readouts to T2* loses.

Fig 6 shows the mean group CBF map of the elderly cohort after standardization to the MNI space. Table 1 summarizes the rest of the parameters extracted from this cohort data. SNR values were comparable to those obtained in Study 2 (performed in the same scanner and with the same head coil), after correcting for the decrease in perfusion signal. A slight reduction in GM-WM ratio was observed in the elderly cohort, likely due to the perfusion losses being more acute in the GM tissue than in the WM.

## Discussion

In this study, we demonstrated the feasibility of using 1D-accelerated 3D readouts to obtain whole-brain, single-shot, high-SNR ASL perfusion maps in both research and clinical populations.

The data obtained with the 1D-accelerated sequence showed increased GM-WM contrast compared to the non-accelerated sequence. This is likely due to the reduced partial volume effects achieved by the decreased effective voxel size in the slice-direction, as predicted by the PSF simulations (Fig 2). As observed in the MTF, the longer readout of the non-accelerated sequence leads to more T_2_ decay, which translates into a wider PSF. This in turn leads to increased through-plane blurring and partial volume effects, as can be observed in Fig 2.

Both sequence versions demonstrated the ability to detect functional activation at the subject level, including cerebellar activity. Activation clusters obtained with the accelerated readout showed a more compact shape, whereas clusters obtained from the non-accelerated data spread more in the slice-direction into the WM tissue (Fig 4), likely as a reflect of the increased through-plane blurring.

No apparent tSNR penalty was found associated with the acceleration scheme employed, despite the increase in effective voxel resolution. The temporal SNR is driven by two factors: the detected signal level and the amplitude of the signal fluctuations. Theory predicts the fluctuations amplitude to be driven by the underlying noise, which has a physiological and a thermal component. The former is approximately proportional to the raw image intensity, while the latter is proportional to the receiver BW (which remains equal across readouts) and to the inverse of the number of coil channels and k-space encodings (which varies in the k_z_ direction). Thus, for a twofold acceleration, an increase factor of √2 in this noise SD component is expected. This value is in very good agreement with the estimated value measured from the data (mean = 1.41, median = 1.34).

On the other hand, a 15% increase in raw signal intensity was observed when acceleration was used. This factor is expected to directly contribute to increase the detected perfusion signal and counteract SNR loss, since physiological noise contributions are assumed to be comparable across readouts due to the use of a background suppression scheme with a high level of suppression (90%).

Additional factors of consideration in the presence of long readouts are subject motion and T_2_ decay. In the presence of motion, shorter readouts are less sensitive to motion-related artifacts, resulting in increased signal temporal stability for accelerated readouts. Secondly, while thermal noise is thought to have a flat power spectrum across the frequency space, the amount of signal detected decreases with time exponentially with T_2_. In the case of long readouts, such as the non-accelerated version used here, it is likely that the signal in the outer part of k-space has decreased below the coil sensitivity level, becoming undetectable, and therefore the contribution of these k-space encodings is limited to thermal noise, resulting in less signal energy present at high frequencies. This is expected to have a negative impact on the image temporal stability, although due to the unknown frequency nature of the physiological noise it is not possible to predict its extent.

An increase in GM-WM ratio and SNR was observed when dividing the single-shot readout into two shots. Although across-study comparisons are limited in this work due to the change in scanner platform, RF coil and subject cohorts, this observation confirms that multi-shot acquisitions can be a useful strategy for improving SNR and further attenuating partial volume effects, when temporal resolution is not a constraint, but at the expense of increased sensitivity to between-shot subject motion [11].

## Conclusions

In conclusion, 3D readouts can be combined with parallel imaging techniques in ASL perfusion MRI to improve image resolution and decrease SAR deposition. In particular, two-fold acceleration factors can be employed to achieve single-shot, whole-brain coverage without any associated loss in the perfusion signal temporal stability, and thus in the statistical power of the detected activations. For studies in which temporal resolution is not paramount, multi-shot 1D-accelerated readouts can further increase resolution, image quality, and SNR. This approach is applicable to both research and clinical populations.

## Supporting information

S1 File Supporting scanning protocol information. (DOCX)

S1 Table Summary of basic demographics among recruited participants for all the studies. SD = Standard deviation.
(DOCX)
