# Supplementary material for: Whole-brain background-suppressed pCASL MRI with 1D-accelerated 3D RARE Stack-Of-Spirals readout
Source: PLoS One. 2017 Aug 24;12(8):e0183762. doi: 10.1371/journal.pone.0183762 (PMC5570334; doi:10.1371/journal.pone.0183762)
Supplement: S1 File — (DOCX) [file pone.0183762.s001.docx]

# S1 Supporting Information

## Materials and Methods

### Scanning protocol

#### Study 1

Imaging parameters of the magnetization prepared rapid gradient echo (MPRAGE) image [1]: Repetition Time (TR) = 1620 ms, Echo Time (TE) = 3.09 ms, Inversion Time (TI) = 950 ms, ﬂip angle = 15°, Field-Of-View (FOV) = 250×187.5×160 mm³, resolution = 0.98×0.98×1 mm³, matrix = 256×192×160, 160 axial slices, scan time = 5.2 min.

Imaging parameters of the time-of-flight (TOF) angiogram [2]: TR/TE = 22/3.86 ms, ﬂip angle = 18°, two slabs of 40 nominal partitions acquired with 20% oversampling and slice partial Fourier (PF) = 6/8, phase PF = 6/8, FOV = 200×150×72 mm³, resolution = 0.52×0.52×1 mm³, matrix = 384×288×72, scan time = 2.88 min.

#### Study 2

Imaging parameters of the MPRAGE image: TR/TE = 1620/3.15 ms, TI = 950 ms, ﬂip angle = 15°, FOV = 250×187.5×160 mm³, resolution = 0.98×0.98×1 mm³, matrix = 256×192×160, 160 axial slices, GRAPPA acceleration factor = 2, reference lines = 24, scan time = 2.9 min.

Imaging parameters of the TOF angiogram: TR/TE = 22/3.66 ms, ﬂip angle = 18°, two slabs of 40 nominal partitions acquired with 15% oversampling and slice partial Fourier (PF) = 6/8, phase PF = 6/8, FOV = 200×150×74 mm³, resolution = 0.52×0.52×1 mm³, matrix = 384×288×74, GRAPPA acceleration factor = 2, reference lines = 24, scan time = 1.63 min.

#### Study 3

All participants were recruited from the Penn Memory Center, a National Institute of Aging supported Alzheimer’s disease center. Participants underwent annual standard medical, neurologic, psychiatric, and neuropsychological evaluations including measures from the National Alzheimer’s Coordinating Center’s (NACC) Uniform Data Set (UDS) [3–5]. The NACC UDS 3.0 includes the Montreal Cognitive Assessment (MoCA), Digit Span Forward & Backward, Trail Making Test, Craft Paragraph Memory Test, Digit Symbol, Verbal Fluency, and Multilingual Naming Test. After initial and annual follow-up, data was reviewed and cases discussed at weekly consensus conferences attended by neurologists, geriatric psychiatrists, geriatric medicine internists, and neuropsychologists. While strict cutoffs were not applied, individuals were considered healthy controls on the basis of both absence of significant memory symptoms (Clinical Dementia Rating=0) and evidence of normal cognitive performance, including a score in the Mini Mental State Examination (MMSE) greater than 27. Other inclusion criteria included age greater than 50, N7 years of education, and English speaking at an early age. Participants were excluded if they had a history of clinical stroke, significant traumatic brain injury, alcohol or drug abuse/dependence, prior electroconvulsive therapy, and any significant disease or medical/psychiatric condition that was felt to impact neuropsychological performance.

Imaging parameters of the MPRAGE image: TR/TE = 2400/2.24 ms, TI = 1060 ms, ﬂip angle = 8°, FOV = 166×240×256 mm³, resolution = 0.8×0.8×0.8 mm³, matrix = 208×300×320, 208 sagittal slices, GRAPPA acceleration factor = 2, reference lines = 32, scan time = 6.6 min.

Imaging parameters of the TOF angiogram: TR/TE = 22/3.66 ms, ﬂip angle = 18°, two slabs of 40 nominal partitions acquired with 15% oversampling and slice partial Fourier (PF) = 6/8, phase PF = 6/8, FOV = 200×150×74 mm³, resolution = 0.52×0.52×1 mm³, matrix = 384×288×74, GRAPPA acceleration factor = 2, reference lines = 24, scan time = 1.63 min.

# References

1. Mugler JP, Brookeman JR. Three-dimensional magnetization-prepared rapid gradient-echo imaging (3D MP RAGE). Magn Reson Med. 1990;15: 152–157. doi:10.1002/mrm.1910150117

2. Haacke EM, Masaryk TJ, Wielopolski PA, Zypman FR, Tkach JA, Amartur S, et al. Optimizing blood vessel contrast in fast three-dimensional MRI. Magn Reson Med. 1990;14: 202–221. doi:10.1002/mrm.1910140207

3. Morris JC, Weintraub S, Chui HC, Cummings J, DeCarli C, Ferris S, et al. The Uniform Data Set (UDS): Clinical and Cognitive Variables and Descriptive Data From Alzheimer Disease Centers. Alzheimer Dis Assoc Disord. 2006;20: 210–216. doi:10.1097/01.wad.0000213865.09806.92

4. Beekly DL, Ramos EM, Lee WW, Deitrich WD, Jacka ME, Wu J, et al. The National Alzheimer’s Coordinating Center (NACC) database: the Uniform Data Set. Alzheimer Dis Assoc Disord. 2007;21: 249–258. doi:10.1097/WAD.0b013e318142774e

5. Weintraub S, Salmon D, Mercaldo N, Ferris S, Graff-radford NR, Chui H, et al. The Alzheimer’s disease centers’ data set (UDS): The neuropsychological test battery. Alzheimer Dis Assoc Disord. 2009;23: 91–101. doi:10.1097/WAD.0b013e318191c7dd.The
