# Supplementary material for: Whole-brain background-suppressed pCASL MRI with 1D-accelerated 3D RARE Stack-Of-Spirals readout
Source: PLoS One. 2017 Aug 24;12(8):e0183762. doi: 10.1371/journal.pone.0183762 (PMC5570334; doi:10.1371/journal.pone.0183762)
Supplement: S1 Table — SD = Standard deviation. (DOCX) [file pone.0183762.s002.docx]

# Supporting S1 Table

**S1 Table. Summary of basic demographics among recruited participants for all the studies**. SD = Standard deviation.

|  | **Number of drop-out / recruited participants** | **Female / Male participant ratio** | **Mean age ± SD [years]** | **Age range [years]** |
| --- | --- | --- | --- | --- |
| **Study 1** | 1 / 6 | 2 / 4 | 35.0 ± 11.5 | 27 – 55 |
| **Study 2** | 0 / 4 | 2 / 2 | 43.0 ± 20.8 | 21 – 65 |
| **Study 3** | 0 / 18 | 12 / 6 | 74.6 ± 8.5 | 61 – 93 |
